# Epidemiology and Geographic Distribution of Blastomycosis, Histoplasmosis, and Coccidioidomycosis, Ontario, Canada, 1990–2015

**DOI:** 10.3201/eid2407.172063

**Published:** 2018-07

**Authors:** Elizabeth M. Brown, Lisa R. McTaggart, Deirdre Dunn, Elizabeth Pszczolko, Kar George Tsui, Shaun K. Morris, Derek Stephens, Julianne V. Kus, Susan E. Richardson

**Affiliations:** Public Health Ontario, Toronto, Ontario, Canada (E.M. Brown, L.R. McTaggart, D. Dunn, E. Pszczolko, K.G. Tsui, J.V. Kus, S.E. Richardson);; University of Toronto, Toronto (E.M. Brown, S.K. Morris, J.V. Kus, S.E. Richardson);; The Hospital for Sick Children, Toronto (S.K. Morris, D. Stephens, S.E. Richardson)

**Keywords:** fungi, dimorphic fungi, climate change, blastomycosis, histoplasmosis, coccidioidomycosis, *Blastomyces dermatitidis*, *Blastomyces gilchristii*, *Histoplasma capsulatum*, *Coccidioides immitis*, *Coccidioides posadasii*, Ontario, Canada, geographic distribution

## Abstract

Endemic mycoses represent a growing public health challenge in North America. We describe the epidemiology of 1,392 microbiology laboratory–confirmed cases of blastomycosis, histoplasmosis, and coccidioidomycosis in Ontario during 1990–2015. Blastomycosis was the most common infection (1,092 cases; incidence of 0.41 cases/100,000 population), followed by histoplasmosis (211 cases) and coccidioidomycosis (89 cases). Incidence of blastomycosis increased from 1995 to 2001 and has remained elevated, especially in the northwest region, incorporating several localized hotspots where disease incidence (10.9 cases/100,000 population) is 12.6 times greater than in any other region of the province. This retrospective study substantially increases the number of known endemic fungal infections reported in Canada, confirms Ontario as an important region of endemicity for blastomycosis and histoplasmosis, and provides an epidemiologic baseline for future disease surveillance. Clinicians should include blastomycosis and histoplasmosis in the differential diagnosis of antibiotic-refractory pneumonia in patients traveling to or residing in Ontario.

## Abstract 2

Elevated incidence of blastomycosis in Ontario calls for diagnostic vigilance.

In North America, the endemic mycoses blastomycosis, histoplasmosis, and coccidioidomycosis are responsible for serious illness in immunocompetent and immunocompromised hosts ranging from asymptomatic, self-limiting illness to invasive, life-threatening disease (*1**,**2*). Infection occurs when a susceptible host inhales fungal spores from the surrounding environment (*2*). Thus, infections occur sporadically, with occasional point-source outbreaks in the localized geographic areas of endemicity defined by the natural habitat of *Blastomyces*, *Histoplasma*, and *Coccidioides* fungi (*2*).

Despite the potential severity of these infections, these diseases are reportable in only select states and provinces, providing only partial coverage of known regions of endemicity (*3*). The lack of mandatory public health reporting in most areas and the small number of epidemiologic studies make it difficult to understand the true burden of disease, which, in turn, contributes to a low clinical index of suspicion, especially outside endemic regions, leading to diagnostic delays and a consequent increase in illness and death (*2**,**4*). Several recent reports suggest increasing incidence and expanding geographic endemicity of the dimorphic fungal infections in North America (*1**,**4**–**10*). Additional shifts in prevalence and endemic range are expected as climate change alters ecosystems in North America (*11*). To address these knowledge gaps and concerns, several more comprehensive epidemiologic assessments have been performed recently in the United States (*12**–**17*).

Although often excluded from disease distribution maps of North America (*18*), the regions to which blastomycosis and histoplasmosis are endemic extend into Canada. Historically, blastomycosis has been considered endemic to Manitoba, northwestern Ontario, and Quebec (*19**–**22*) with the Kenora area of northwestern Ontario exhibiting the highest reported incidence of blastomycosis in the world (*4**–**6**,**23*). Before 1989, when mandatory reporting in Ontario was suspended, cases of blastomycosis were rare (1.8 cases/year) and thought to be acquired almost exclusively in the northwest region of the province (*24*). Since that time, the known blastomycosis-endemic range has expanded to include all of Ontario; provincial incidence increased until 2003 or later (*4**,**25*)*.* A recent study in Quebec confirms the endemic status of blastomycosis (*26*); sporadic clusters of human and canine infections have occurred in Saskatchewan (*27*) and New Brunswick (*19*). Histoplasmosis is considered endemic to regions bordering the St. Lawrence River (*19**,**28**–**30*), especially Quebec (*19**,**31**,**32*); a single case cluster occurred in Alberta (*19*), but there are no recent epidemiologic reports from Ontario. Coccidioidomycosis is not considered endemic to Canada, but data on travel-related cases are outdated (*19*).

With approval from Research Ethics committees at Public Health Ontario and The Hospital for Sick Children, we describe the epidemiology of microbiology laboratory–confirmed cases of blastomycosis, histoplasmosis, and coccidioidomycosis in Ontario, Canada, during 1990–2015. When combined with studies from Manitoba (*21*) and Quebec (*26**,**32*), this study provides a more comprehensive picture of the incidence of blastomycosis and cases of histoplasmosis from mycosis-endemic regions in Canada to complement US studies. 

## Methods

### Study Setting, Data Sources, and Case Definition

**Figure 1:**
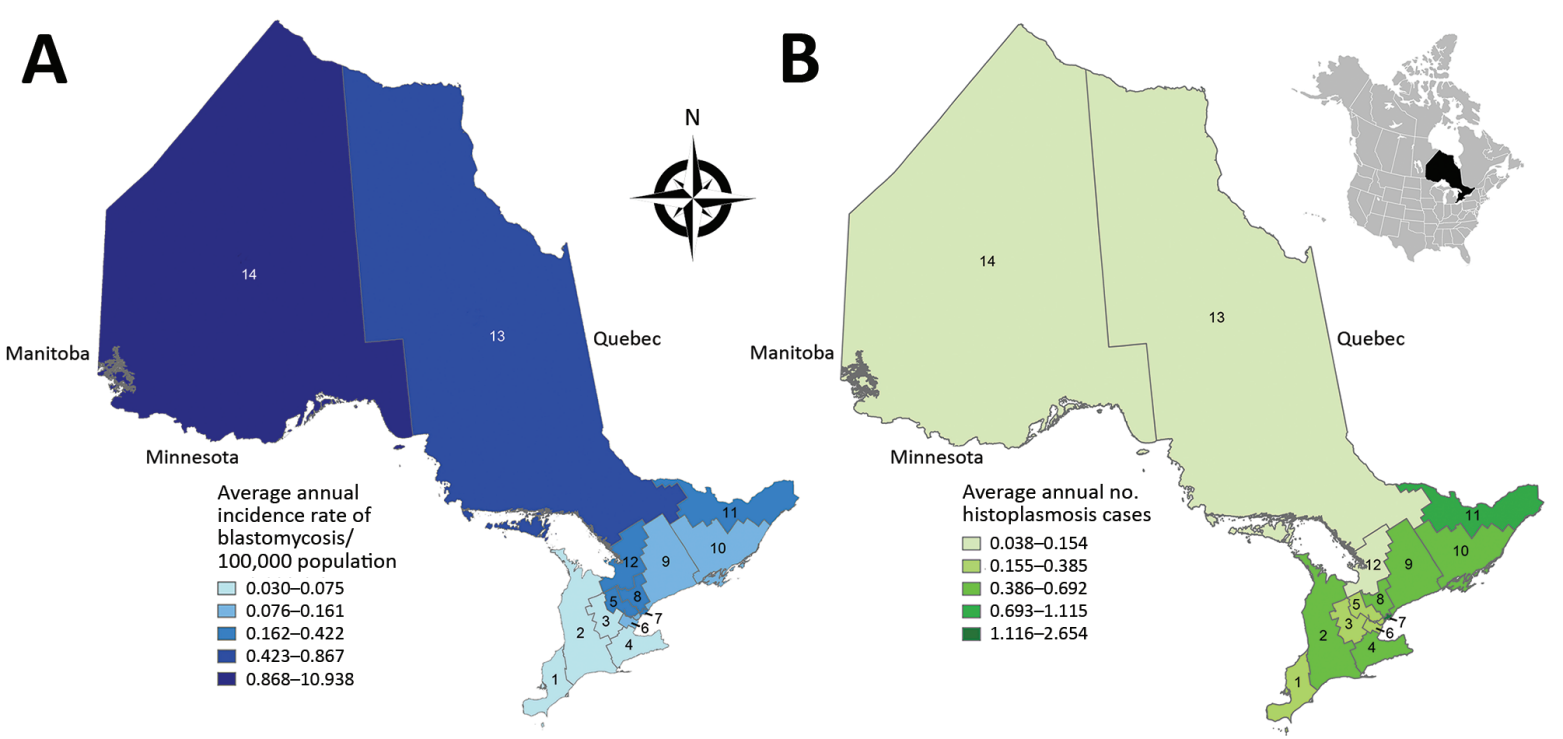
Geographic distribution of A) annualized incidence (no. cases/100,000 population) of blastomycosis (1995–2015) and B) no. cases of histoplasmosis (1990–2015) by Ontario Local Health Integration Network (LHIN), Ontario, Canada. 1, Erie St. Clair; 2, South West; 3, Waterloo Wellington; 4, Hamilton Niagara Haldimond Brant; 5, Central West; 6, Mississauga Halton; 7, Toronto Central; 8, Central; 9, Central East; 10, South East; 11, Champlain; 12, North Simcoe Muskoka; 13, North East; 14, North West. Incidence was calculated using LHIN-specific population denominators from Statistics Canada (*36*). Inset shows the location of Ontario within North America.

Ontario, Canada’s most populous province (population of 13.4 million in 2016 [*33*]), is divided into 14 Local Health Integration Networks (LHINs) that provide health services for their respective populations (Figure 1, panels A, B). Because of the need for specialized expertise and containment level 3 laboratory facilities for manipulating *Histoplasma*, *Blastomyces,* and *Coccidioides* species, Public Health Ontario Laboratory (PHOL) is the only referral facility in the province for their handling and diagnosis. This centralization ensures a high level of provincewide case ascertainment for microbiology laboratory–confirmed human infections. We performed a retrospective review of PHOL data to detect cases of blastomycosis, histoplasmosis, and coccidioidomycosis. Inclusion criteria were positive culture, microscopy, or both for *Blastomyces dermatitidis/gilchristii* during January 1, 1995–December 31, 2015; for *Coccidioides immitis/posadasii* and *Histoplasma capsulatum* infections, the study period was January 1, 1990–December 31, 2015. Patients with >1 specimen submitted within a 6-month period were each counted as a single case. Demographic information included patient age, sex, and address (city, forward sortation area [FSA, first 3 characters of postal code]; sender address (institution, city, FSA); date of specimen receipt; and specimen type. When specific data were not available, we excluded cases from individual analyses requiring these data (Table 1). We assessed statistical significance by χ^2^ test (p≤0.05 was statistically significant).

**Table 1:** Characteristics of microbiology laboratory–confirmed blastomycosis, histoplasmosis, and coccidioidomycosis cases reported in Ontario, Canada, 1990–2015

Characteristic	No. (%) cases*		
	Blastomycosis, n = 1,092	Histoplasmosis, n = 211	Coccidioidomycosis, n = 89
Patient sex	n = 963	n = 180	n = 80
M	627 (65.1)	144 (80.0)	48 (60.0)
F	336 (34.9)	36 (20.0)	32 (40.0)
Patient age, y	n = 973	n = 158	n = 71
<19	126 (12.9)	2 (1.3)	0
20–29	119 (12.2)	11 (6.7)	1 (1.4)
30–39	167 (17.2)	28 (17.7)	7 (9.9)
40–49	201 (20.7)	32 (20.3)	7 (9.9)
50–59	175 (18.0)	47 (29.7)	22 (31.0)
60–69	90 (9.2)	22 (13.9)	21 (29.6)
>70	95 (9.8)	16 (10.1)	13 (19.7)
Source of specimen isolation	n = 895	n = 202	n = 81
Respiratory	754 (84.2)	91 (45.0)	65 (80.2)
Skin, wound, subcutaneous tissue	77 (8.6)	14 (6.9)	5 (6.2)
Mucous membrane†	6 (0.67)	3 (1.5)	0
Bone, joint	14 (1.6)	2 (0.99)	3 (3.7)
Genitourinary	1 (0.11)	0	0
Gastrointestinal	2 (0.22)	7 (3.5)	0
CNS	6 (0.67)	7 (3.5)	0
Other‡	10 (1.1)	46 (22.8)	3 (3.7)
Multiple§	25 (2.8)	32 (15.8)	5 (6.2)

*Counts for blastomycosis are from 1995–2015 and for histoplasmosis and coccidioidomycosis from 1990–2015 data. We omitted cases for which age, sex, or source of specimen isolation were unknown from the calculations. n values by each category are also provided. †Specimen types included ocular fluid, oral biopsy (tongue), nasal swab, and nasal biopsy. ‡Specimen types included bone marrow, lymph node tissue, blood, parathyroid gland tissue, and adrenal gland tissue and fluid. §Specimens from >2 noncontiguous body sites received <6 months apart.

### Descriptive Epidemiologic Analysis

We calculated annual and stratum-specific (age-, LHIN-, and regional group–specific) incidence (no. cases/100,000 population) for blastomycosis using population denominators from Statistics Canada extracted from the Ontario Ministry of Health and Long-Term Care: IntelliHealth Ontario on February 18, 2014, and January 15, 2016. We used population projections for 2014 and 2015 (*34*).

We examined temporal trends in disease occurrence by performing aggregated seasonal case counts based on date of specimen receipt (date of symptom onset was not available). We defined winter as December–February, spring as March–May, summer as June–August, and autumn as September–November (*35*). We assessed significance by χ^2^ test (Bonferroni-corrected p<0.05 was statistically significant).

### Geographic Distribution, Spatial Statistics, and Hotspot Analysis

We examined the geographic distribution of blastomycosis and histoplasmosis by assigning each case to 1 of Ontario’s 14 LHINs. We used the patient’s home address, if known, to assign the case to a LHIN (blastomycosis n = 544, histoplasmosis n = 42). If the patient’s home address was not known (blastomycosis n = 526, histoplasmosis n = 169), we used the sender’s (i.e., hospital, physician’s office, or community health center) FSA to assign cases to LHINs. Of 586 cases in which both patient’s home FSA and sender’s FSA were known, 89.3% (523/586) of the time they were the same, suggesting that sender’s FSA is a usable surrogate for patient location. Patient and sender location were unknown for 22 cases of blastomycosis. We mapped annualized incidence rates of blastomycosis and number of cases of histoplasmosis across Ontario’s 14 LHINs using ArcGIS version 10.4 software (ESRI Inc., Redlands, CA, USA). We obtained Ontario and LHIN boundary files from Statistics Canada (*36*).

To examine temporal and geographic trends for blastomycosis, we aggregated data from the LHINs into 5 larger regional groups by geographic continuity and similar incidence rates: Northwest (North West LHIN); Northeast (North East LHIN); South-central (Toronto, North Simcoe Muskoka, Central, and Central West LHINs); Southeast (Central East, South East, and Champlain LHINs); and Southwest (Erie St. Clair, South West, Waterloo Wellington, Hamilton Niagara Haldimand Brant, and Mississauga Halton LHINs). Because some of the LHINs had very few cases, we aggregated data to stabilize the variance from data with sparse cells. We applied the GENMOD procedure in SAS software version 9.4 (SAS Institute Inc., Cary, NC, USA), to categorize data into the 5 geographic regions and 4 time intervals (1995–1999, 2000–2004, 2005–2009, 2010–2015). We fitted data to Poisson regression models with the logarithm of total population within each time interval and region used as an offset. We calculated incidence rate ratios (IRR) and 95% CIs by performing a series of pairwise contrast estimates between each regional group and time interval (p<0.05 was statistically significant). We further investigated temporal changes within each regional group using 6 pairwise comparisons of annual incidence between each of the 4 time intervals with a Tukey-Kramer adjustment for multiple comparisons.

We conducted spatial analysis with clustering methods to identify hotspots of blastomycosis using Spatial Statistics Toolbox Getis-Ord Gi* statistic in ArcGIS version 10.4. We performed optimized hotspot analysis using case counts normalized with 2016 census subdivision data from Statistics Canada (*33**,**37*). We set polygons to Statistics Canada census subdivisions with polygons with “0” incidences included in the analysis. Statistically significant spatial clustering of higher than average (hotspot) and lower than average (coldspot) values were identified at CIs 90%, 95%, and 99% (1 − p value), signifying the intensity of the hotspot or coldspot. We restricted analysis to cases for which patient home city, FSA, or both were available (n = 544). We plotted individual cases by patient home city, FSA, or both, with circle size proportional to number of cases.

## Results

We identified 1,392 laboratory-confirmed dimorphic fungal infections in Ontario during 1990–2015. Among these, blastomycosis was the most common (n = 1,092; 78.4%), followed by histoplasmosis (n = 211; 15.2%) and coccidioidomycosis (n = 89; 6.4%).

### Blastomycosis

**Figure 2:**
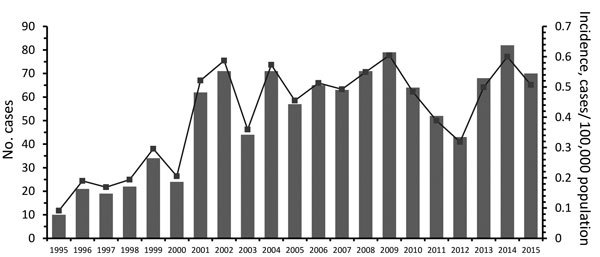
The number of cases (bars) and annual incidence (line) of microbiology laboratory–confirmed blastomycosis in Ontario, Canada, 1995–2015. Incidence was calculated using population denominators from Statistics Canada (*36**,**37*).

During the study period, a median of 62 cases/year (range 10–82 cases/year) of blastomycosis occurred; yearly incidence ranged from 0.09–0.60/100,000 population, with an overall annual incidence rate of 0.41/100,000 population (95% CI 0.31–0.52) (Figure 2). Men were more frequently infected than women (p<0.001), and infection was most common in those 40–49 years of age (Table 1). Pediatric patients (<19 years of age) represented 12.9% of cases; 2 cases were reported in infants <1 year old. *Blastomyces* fungus was most commonly isolated from respiratory specimens, followed by skin, wounds, subcutaneous tissue, and bone/joint (Table 1). We observed seasonal trends; significantly more cases were diagnosed in the autumn (Bonferroni-corrected p = 0.002) and winter (Bonferroni-corrected p = 0.024) than summer (Technical Appendix Figure 1).

The incidence of blastomycosis in Ontario increased from 0.09/100,000 population in 1995 to 0.52/100,000 population in 2001 and then remained elevated during 2001–2015 (0.48/100,000 population), peaking in 2009 and 2014 with annual incidence rates of 0.60/100,000 population (Figure 2). This increase was statistically significant as indicated by Poisson regression IRRs comparing 1995–1999 versus 2000–2004, 2005–2009, and 2010–2015 (Table 2). Geographic regional analysis suggested that this increase was largely attributable to the Northwest region of the province, where there was also a statistically significant increase in blastomycosis during the same time intervals (Table 2; Technical Appendix Figure 2). We detected no significant temporal trends in any of the other geographic regions.

**Table 2:** Temporal and geographic trends of annual incidence and incidence rate ratios of blastomycosis in Ontario, Canada, 1990–2015, by province and region*

Geographic region	Years	Annual incidence	Poisson regression analysis, IRR (95% CI)†					
			Ontario					
			1995–1999		2000–2004		2005–2009	
Ontario	1995–1999	0.19						
	2000–2004	0.42	**3.62 (1.91–6.86)**					
	2005–2009	0.52	**4.46 (2.39–8.35)**		1.23 (0.82–1.84)			
	2010–2015	0.47	**3.87 (2.05–7.31)**		1.07 (0.71–1.62)		0.87 (0.58–1.29)	
			Northwest					
			1995–1999		2000–2004		2005–2009	
Northwest	1995–1999	1.91						
	2000–2004	14.60	**7.31 (2.87–18.64)**					
	2005–2009	15.49	**7.91 (3.11–20.10)**		1.07 (0.68–1.69)			
	2010–2015	11.90	**6.12 (2.37–15.82)**		0.83 (0.51–1.35)		0.77 (0.48–1.25)	
			Southwest	South-central		Southeast		Northeast
Southwest	1995–2015	0.05						
South-central	1995–2015	0.29	**3.09 (1.01–9.50)**					
Southeast	1995–2015	0.14	2.00 (0.62–6.49)	0.65 (0.28–1.52)				
Northeast	1995–2015	0.87	**8.24 (2.82–24.12)**	**2.66 (1.32–5.35)**		**4.12 (1.88–9.07)**		
Northwest	1995–2015	10.9	**105.21 (38.78–285.43)**	**33.97 (19.10–60.41)**		**52.68 (26.69–104.00)**		**12.76 (7.92–20.56)**

*Incidence is no. cases/100,000 population. IRR, incidence rate ratio.  †IRRs were derived from Poisson regression analysis showing pairwise contrasts of blastomycosis incidence rates between different time intervals and geographic regions. Bold type indicates statistically significant values (p<0.05).

**Figure 3:**
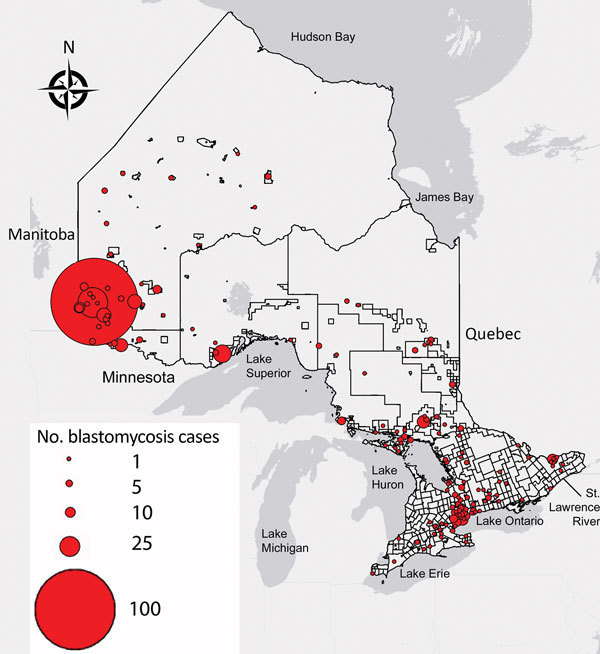
Geographic distribution of blastomycosis cases with known patient city and forward sortation area (first 3 characters of postal code) (n = 544) in Ontario, 1995–2015. Size of dot is proportional to number of cases at a given location.

The incidence of blastomycosis varied considerably across provincial LHINs (Figure 1, panel A). Disproportionately more cases of blastomycosis were from the North West LHIN (51.3%, n = 560), where the annualized incidence of 10.9/100,000 population (Figure 1, panel A) was 12.6 times greater than any other LHIN. Poisson regression analysis contrasting regional groups showed that the rate of infection was 12.8–105.2 times greater in the Northwest region compared with all other groups (Table 2). Several statistically significant hotspots (95%–99% CI) were identified in and around Kenora and Rainy River, Ontario, located in the Northwest region (Technical Appendix Figure 3), consistent with the large number of cases in this area (Figure 3). Rates of disease were also significantly elevated in the Northeast region (0.87/100,000 population) compared with the 3 lower-incidence southern regions (Southeast, South-central, Southwest) (Table 2). We identified no statistically significant coldspots (Technical Appendix Figure 3). In addition to the high number of cases in the Northwest and Northeast regions, we saw a substantial distribution of blastomycosis cases extending into the South-central region (including the Toronto area) during the study period (Technical Appendix Figure 2; Figure 3).

### Histoplasmosis

**Figure 4:**
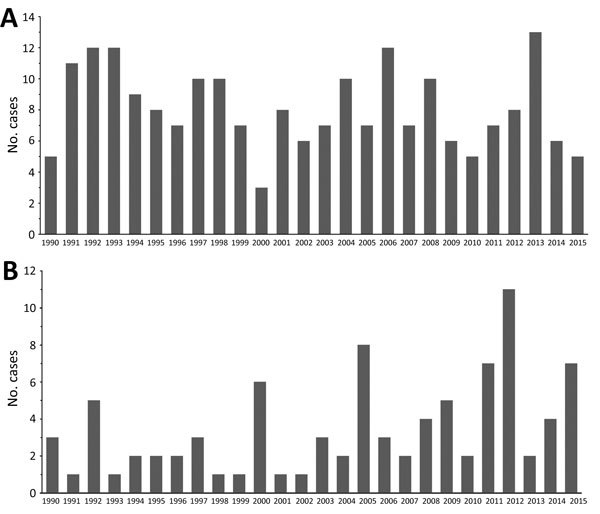
The number of cases of microbiology laboratory–confirmed A) histoplasmosis and B) coccidioidomycosis in Ontario, Canada, 1990–2015.

There were 211 cases of laboratory-confirmed cases of histoplasmosis in Ontario (1990–2015), but no year-on-year or seasonal trends were observed (Figure 4, panel A; Technical Appendix Figure 1). We identified a median of 7.5 cases each year (range 3–13 cases/year). A diagnosis of histoplasmosis was more common in men than women (p≤0.001); the greatest proportion of cases occurred in the 50–59 year-old cohort, incorporating both sexes (47/158; 29.7%). Respiratory specimens represented almost half (45%) of the cases, followed by skin, wound, subcutaneous tissue (6.9%), bone marrow (8.9%), and lymph node tissue (7.4%) (Table 1). By geographic distribution, histoplasmosis cases were concentrated in the Toronto Central (69 cases), South East (18 cases), and Champlain (29 cases) LHINs (Figure 1, panel B).

### Coccidioidomycosis

For 1990–2015, we detected 89 cases of coccidioidomycosis, a median of 2.5 cases/year (range 1–11 cases/year). We observed no year-on-year or seasonal trends in disease occurrence, yet case counts were notably higher in 1992, 2000, 2005, 2011, 2012, and 2015 (Figure 4, panel B; Technical Appendix Figure 1). As observed for the other endemic mycoses, men were more frequently infected (p<0.001) (Table 1). Median patient age was 59 years (range 24–90 years), and the greatest proportion of cases occurred in the 50–59 (22/71; 31.0%) and 60–69 (21/71; 29.6%) year-old cohorts. Respiratory specimens were the most common source of isolates (80.2%) (Table 1).

## Discussion

Our 26-year longitudinal study characterized the epidemiology of microbiologically confirmed cases of blastomycosis, histoplasmosis, and coccidioidomycosis in Ontario, Canada. Although we underestimated the true burden of these diseases by not capturing non–culture-based diagnoses (confirmed through serology, histopathology, or antigen testing), these data substantially increase the known number of cases of endemic fungal infections reported in Canada. Clinicians and public health officials need to be aware that Ontario represents an important region of endemicity for blastomycosis and histoplasmosis and should consider these infections in their differential diagnoses, especially in cases of pneumonia that fails to respond to empiric antimicrobial drugs, in patients residing in or traveling to Ontario, Canada.

Blastomycosis represents an increasingly substantial public health concern in Ontario. The annualized incidence, determined from microbiologically confirmed cases for the province (0.41 cases/100,000 population), is higher than that previously reported for 1994–2003 (0.3 cases/100,000 population) (*4*). The incidence within individual LHINs is also increasing. Morris et al. (*4*) noted that disease rates in Ontario increased from 1.8 cases/year during 1981–1989, when blastomycosis was a reportable disease in Ontario, to 59 cases/year in 2001–2003, when it was no longer reportable. We confirm a statistically significant increase in blastomycosis from the late 1990s (0.19 cases/100,000 population) to the early 2000s (0.42 cases/100,000 population) and further show that the incidence remained elevated until 2015 (0.52 cases/100,000 population for 2005–2009 and 0.47 cases/100,000 population for 2010–2015). Most of this effect was attributable to the ≈6- to 7-fold increase in incidence in northwestern Ontario during the corresponding time intervals. Laboratory practices for culture isolation and identification have not changed over the study period; however, enhanced public awareness in the late 1990s may have facilitated more diagnoses (*4**,**6*).

The provincial and North West LHIN rates of blastomycosis are probably underestimated because they do not include cases identified solely by histopathology or serology or those identified outside the province. A substantial number of cases from northwestern Ontario are diagnosed in the bordering province of Manitoba (59/143 Ontario cases, 41.3%, during 1988–1999) (*4**,**5**,**21*). Few cases are diagnosed by antigen testing, which is not performed in Ontario. For 2006–2015, Litvenjenko and Lunny reported 581 blastomycosis hospitalizations in Ontario (0.44 cases/100,000 population), which included cases identified by nonculture methods but not nonhospitalized patients (*23*). By comparison, we identified more microbiology laboratory–confirmed cases (n = 657) during the same time (0.50 cases/100,000 population), suggesting that laboratory counts at PHOL do provide a high degree of case ascertainment of blastomycosis in Ontario. Among the Canadian provinces of Manitoba, Ontario, and Quebec, which are endemic for blastomycosis, Manitoba reported the highest rate of 0.62 cases/100,000 population (1988–1999) (excluding Ontario residents treated in Manitoba [*21*]). Quebec reported a much lower overall rate of 0.13/100,000 population (1988–2011) (*26*).

Given the seriousness of blastomycosis and the consistently elevated incidence, we have advocated in the past for the reinstatement of mandatory disease reporting. Recent legislative changes passed in December 2017 have designated blastomycosis as a communicable disease reportable to public health authorities in Ontario (*38*). Timely access to comprehensive surveillance data will allow for a more accurate assessment of disease incidence. It will enable public health officials to track changes in disease incidence or regions of endemicity caused by anthropogenic activities and climatic changes and disturbances (*1**,**11*), and to identify case clusters and point-source outbreaks. Mandatory disease reporting and surveillance will aid the diagnosis of unknown cases, enable prompt initiation of treatment to decrease illness and death (*2**,**7**,**39*), and provide support for targeted public health interventions, such as public awareness campaigns (e.g., health advisories for blastomycosis in Big Grassy First Nation and Manitoulin Island, Ontario) (*6**,**7**,**40**,**41*) and preventive measures for vulnerable groups.

This study reaffirms that the Northwest region of Ontario is highly endemic for blastomycosis with an increasing incidence of the disease over the study period. The North West LHIN incidence of 10.9 cases/100,000 population is substantially higher than the provincial rate of 0.41 cases/100,000 population. The Northwestern Health Unit (western half of the North West LHIN) has a hospitalization rate for blastomycosis of 35.0/100,000 population (*23*), whereas the Kenora area is reportedly hyperendemic with an incidence of 117.2 cases/100,000 population *(**6**)* and a hospitalization rate of 57.9/100,000 population (*23*). Our analysis also shows several hotspots of blastomycosis in and around the cities of Kenora and Rainy River, with a correspondingly high number of cases of blastomycosis in nearby northern counties of Minnesota (*42*). These hotspots should be interpreted as intersections between areas of human habitation and an ecologic niche in which the conditions promote fungal growth, liberation, and subsequent host infection. The Eagle River area of Wisconsin is a similar localized blastomycosis-hyperendemic region (100 cases/100,000 population) (*43*), with blastomycosis endemic to a much larger geographic area encompassing the US states bordering the Mississippi and Ohio rivers (*14*). The Northeast region of Ontario had the second highest incidence (0.87 cases/100,000 population) in Ontario, followed by the South-central region, which includes Toronto (0.29 cases/100,000 population). Whereas some of the infections may have been acquired during travel to northwestern Ontario, physicians are increasingly encountering patients with blastomycosis who have not traveled to high-incidence locales (*25**,**44**,**45*), suggesting an increased, although statistically unsupported, environmental presence of *Blastomyces* spp. in the Northeast and South-central regions of the province.

Similar to other studies (*4**,**5**,**35*), we observed seasonality of blastomycosis. This finding suggests summer exposure followed by a variable incubation period of 30–45 days (up to 106 days) (*39**,**46*), resulting in diagnosis in the autumn and winter months.

There were 211 microbiology laboratory–confirmed histoplasmosis cases in Ontario from 1990–2015. *H. capsulatum* is endemic to the states along the Mississippi River basin and the regions bordering the St. Lawrence Seaway and Great Lakes River Drainage Basins (*12**,**15**,**19**,**30*). Whereas there are a few older reports of histoplasmosis in Ontario (*19**,**28**–**30*) and Quebec (*19**,**31**,**32*), this study reaffirms Ontario as an area of endemicity. Consistent with its known epidemiologic range, we observed the highest proportion of cases of histoplasmosis in the LHINs bordering the Great Lakes and the St. Lawrence Seaway. Given these findings, we recommend further study to determine the true incidence of histoplasmosis in Ontario; studies should incorporate not only microbiology laboratory–confirmed cases but also those identified by other common diagnostic modalities, such as serology, antigen testing, and histopathology. Frequent isolation from nonrespiratory specimens (e.g., lymph tissue, nodes, and bone marrow) is consistent with lymphohematogenous spread during infection (*47*) but also suggests that pulmonary mycoses are underrepresented among culture-confirmed cases in Ontario, presumably because they are diagnosed by nonculture methods.

Coccidioidomycosis is not endemic to Canada, and any cases diagnosed in Canada are considered to have been acquired during travel to coccidioidomycosis-endemic areas (*19**,**48*) specifically the southwestern United States, northern Mexico, and parts of Central and South America (*2*). Although patient travel history was not included in this study, the low number of cases of coccidioidomycosis (n = 89) support this conclusion. Previous Canadian studies report only 2 cases in Ontario (*19**,**48*). We report 89 cases (2.5 cases/year), a substantial increase that may be caused by an increase in travel of retirees or others to areas endemic to or experiencing an increased incidence of disease (*2**,**13**,**15**,**17**,**19*). In Ontario, peaks in disease incidence for 2005, 2011, and 2015 mirrored those in California and Arizona (*13**,**49*). Thus, physicians should consider coccidioidomycosis as a potential cause of disease when treating patients with appropriate symptoms and a history of travel to the southwestern United States.

As with any retrospective study, limitations are inherent to the design. We did not capture symptomatic and mild self-limiting infections, which represent a large proportion of all infections (50%–90%, depending on the fungus) (*2*). Likewise, we did not include mycoses treated empirically without microscopy or culture proof, cases identified at autopsy that did not undergo culture (≈33% of CNS blastomycosis cases [*50*]), cases confirmed solely through histopathology or serology, or cases diagnosed outside Ontario (*21*). We did not genotype repeat isolates from the same patient to investigate persistence or reactivation of the disease. Overall, the numbers presented in this analysis most likely underestimate the true extent of these infections in Ontario. Even though patient demographics were missing for some cases, our results were akin to those reported in other jurisdictions (*14**,**15**,**17*). We calculated incidence on the basis of cases assigned to LHINs using patient home address FSA or hospital or physician FSA, which may or may not represent where the infection was acquired (*4*).

In conclusion, this work contributes substantially to our understanding of the geographic distribution and epidemiology of the dimorphic endemic mycoses in Ontario, Canada; however, many cases have likely been missed. The recent restoration of blastomycosis to the list of public health–reportable diseases will assist outbreak investigation, public health planning, and patient and physician education.

Technical Appendix Additional information about the distribution of blastomycosis, histoplasmosis, and coccidioidomycosis in Ontario, 1990–2015.
