# Supplementary material for: Epidemiology and Geographic Distribution of Blastomycosis, Histoplasmosis, and Coccidioidomycosis, Ontario, Canada, 1990–2015
Source: Emerg Infect Dis. 2018 Jul;24(7):1257–66. doi: 10.3201/eid2407.172063 (PMC6038754; doi:10.3201/eid2407.172063)
Supplement: Technical Appendix — Additional information about the distribution of blastomycosis, histoplasmosis, and coccidioidomycosis in Ontario, 1990–2015. [file 17-2063-Techapp-s1.pdf]

Article DOI: https://doi.org/10.3201/eid2407.172063

Epidemiology and Geographic Distribution of Blastomycosis, Histoplasmosis, and Coccidioidomycosis, Ontario, Canada, 1990–2015

Technical Appendix

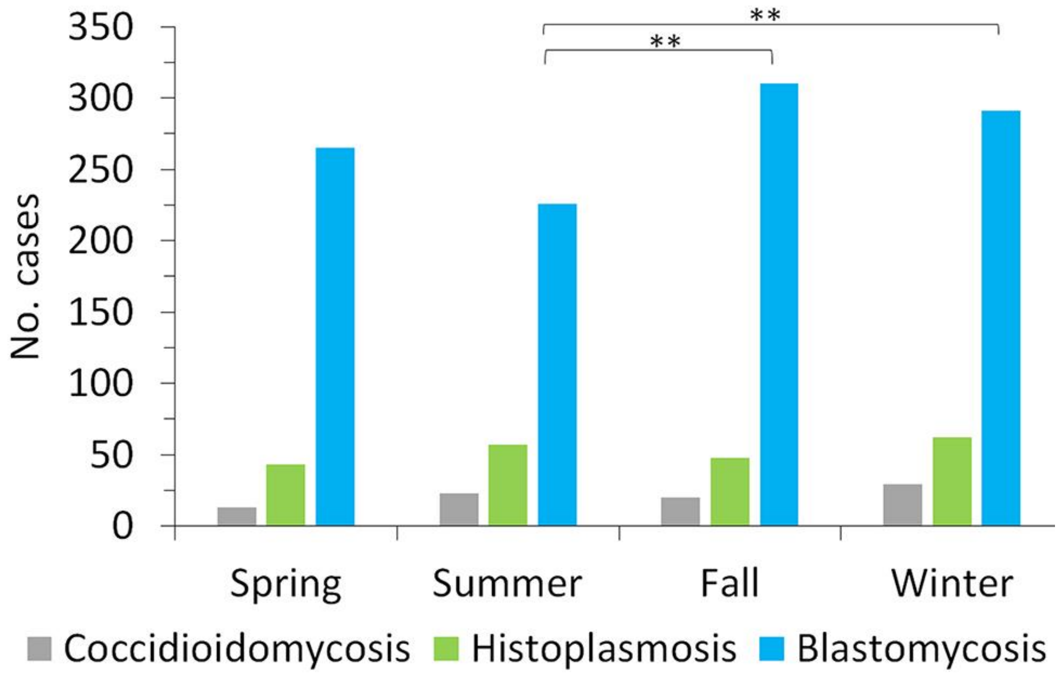

Technical Appendix Figure 1. Seasonal distribution of microbiology laboratory-confirmed blastomycosis, histoplasmosis and coccidioidomycosis cases in Ontario, 1990–2015, by season: spring (March–May), summer (June–August), autumn (September–November), and winter (December–February). **The number of cases diagnosed in autumn (Bonferroni-corrected p = 0.002) and winter (Bonferroni-corrected p = 0.024) were statistically significant compared to the number diagnosed during the summer.

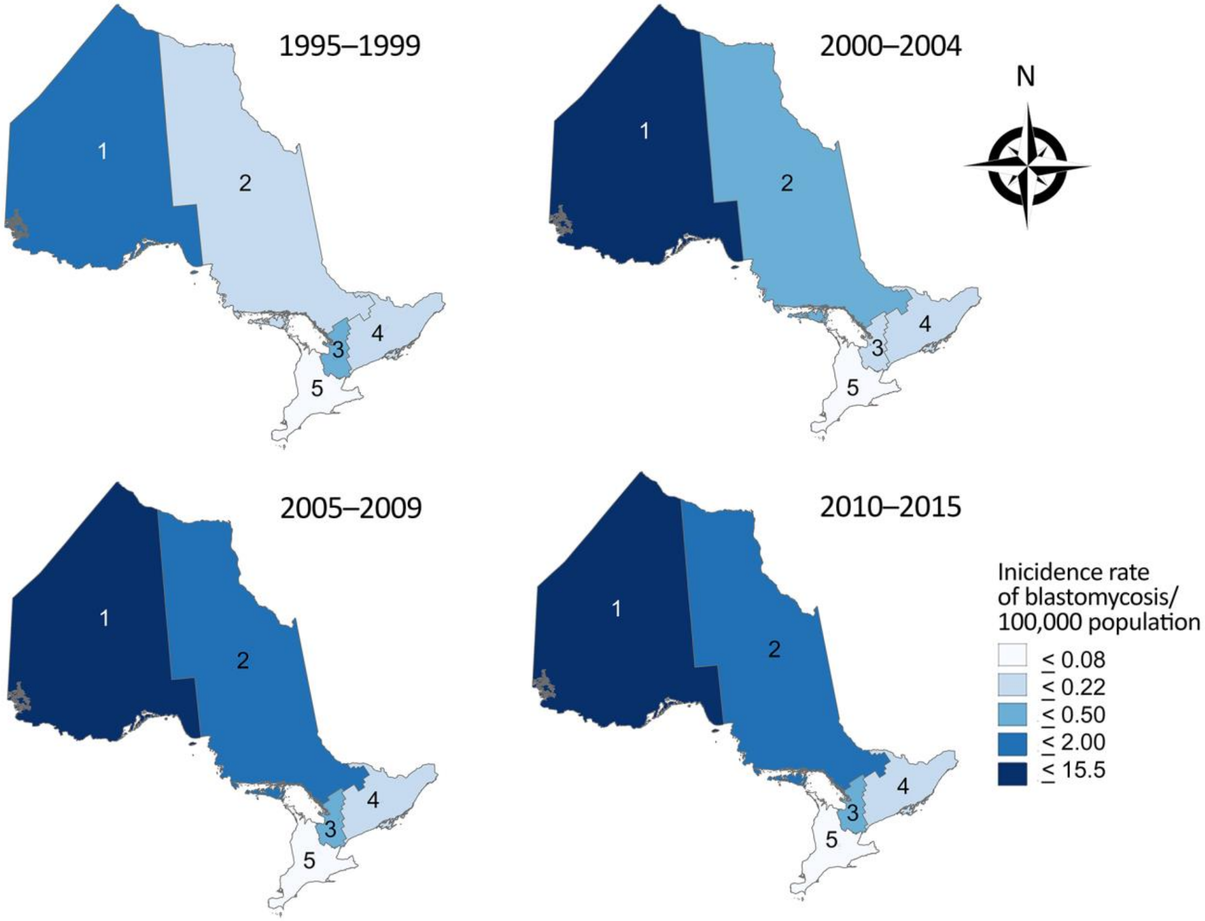

Technical Appendix Figure 2. Temporal trends of incidence (no. cases/100,000 population) of blastomycosis by geographic region. Incidence was calculated by geographic region using population denominators from Statistics Canada for 1995–1999, 2000–2004, 2005–2009, and 2010–2015. 1, Northwest; 2, Northeast; 3, South-central; 4, Southeast; 5, Southwest.

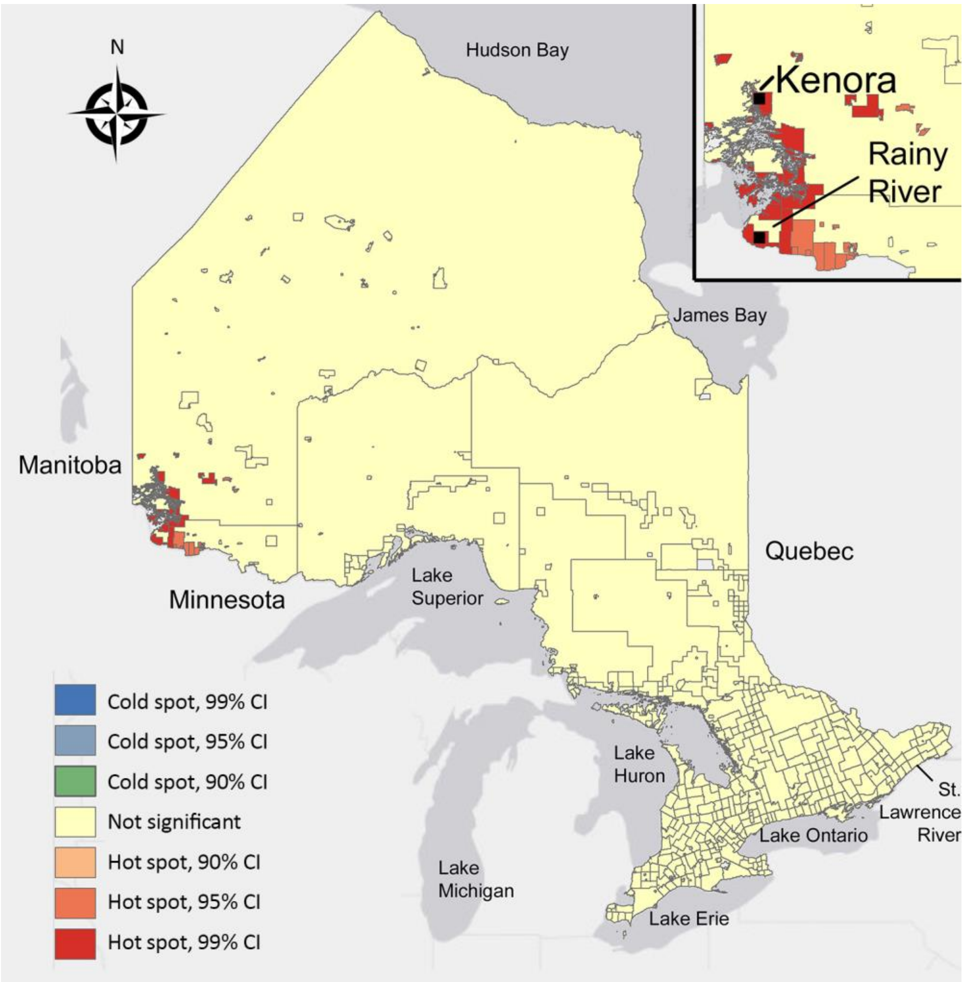

Technical Appendix Figure 3. Hotspot analysis of blastomycosis cases with known patient city and FSA (n = 544) in Ontario from 1995–2015 normalized by population denominators from Statistics Canada and partitioned by census subdivision. Confidence intervals (90%, 95%, and 99%) signify the intensity of aggregation of hotspots or coldspots. Several hotspots were detected surrounding Kenora and Rainy River, Ontario (inset).
